# Comparison of 270-degree percutaneous transforaminal endoscopic decompression under local anesthesia and minimally invasive transforaminal lumbar interbody fusion in the treatment of geriatric lateral recess stenosis associated with degenerative lumbar spondylolisthesis

**DOI:** 10.1186/s13018-023-03676-x

**Published:** 2023-03-09

**Authors:** Yubo Li, Xiaokang Cheng, Bin Chen

**Affiliations:** 1 Department of Minimally Invasive Spine Surgery, The Affiliated Hospital of Chengde Medical University, Chengde, 067000 Hebei China; 2 Department of Orthopedics, Beijing Tongren Hospital, Capital Medical University, Beijing, 100730 China

**Keywords:** Degenerative lumbar spondylolisthesis, Lateral recess stenosis, Percutaneous transforaminal endoscopic decompression, Minimally invasive transforaminal lumbar interbody fusion, Geriatric

## Abstract

**Purpose:**

Various lumbar decompression techniques have been used for the treatment of degenerative lumbar spondylolisthesis (DLS). Few studies have compared the clinical efficacy of percutaneous transforaminal endoscopic decompression (PTED) and minimally invasive transforaminal lumbar interbody fusion (MIS-TLIF) in the treatment of lateral recess stenosis associated with DLS (LRS-DLS) in geriatric patients. The objective of the study was to compare the safety and short-term clinical efficacy of 270-degree PTED under local anesthesia and MIS-TLIF in the treatment of LRS-DLS in Chinese geriatric patients over 60 years old.

**Materials and methods:**

From January 2017 to August 2019, the data of 90 consecutive geriatric patients with single-level L4-5 LRS-DLS were retrospectively reviewed, including those in the PTED group (*n* = 44) and MIS-TLIF group (*n* = 46). The patients were followed up for at least 1 year. Patient demographics and perioperative outcomes were reviewed before and after surgery. The Oswestry Disability Index (ODI), visual analog scale (VAS) for leg pain, and modified MacNab criteria were used to evaluate the clinical outcomes. X-ray examinations were performed 1 year after surgery to assess the progression of spondylolisthesis in the PTED group and bone fusion in the MIS-TLIF group.

**Results:**

The mean patient ages in the PTED and MIS-TLIF groups were 70.3 years and 68.6 years, respectively. Both the PTED and MIS-TLIF groups demonstrated significant improvements in the VAS score for leg pain and ODI score, and no significant differences were found between the groups at any time point (*P* > 0.05). Although the good-to-excellent rate of the modified MacNab criteria in the PTED group was similar to that in the MIS-TLIF group (90.9% vs. 91.3%, *P* > 0.05), PTED was advantageous in terms of the operative time, estimated blood loss, incision length, drainage time, drainage volume, length of hospital stay, and complications.

**Conclusions:**

Both PTED and MIS-TLIF led to favorable outcomes in geriatric patients with LRS-DLS. In addition, PTED caused less severe trauma and fewer complications. In terms of perioperative quality-of-life and clinical outcomes, PTED could supplement MIS-TLIF in geriatric patients with LRS-DLS.

## Introduction

Degenerative lumbar spondylolisthesis (DLS), also known as “low-grade rigid” spondylolisthesis, is the forward translation of a vertebral body with respect to the inferior vertebral body [1]. Patients may present with back pain, radicular leg pain, and pseudoclaudication. It is generally believed that if conservative treatment fails, then surgical treatment should be considered [2]. Hitherto, there has been a continuing debate on the treatment for LSS with DLS [3]. Both decompression alone and decompression with fusion have been used to treat LSS with DLS, especially in recent years [4]. Some researchers [5] have concluded that for the quality of life of individuals with DLS, the addition of interbody fusion leads to greater improvement than decompression alone. Others [6, 7] have suggested that the addition of fusion is not more beneficial in patients with DLS. All of above trials, which compare decompression alone and decompression with fusion, show that the choice of fusion or non-fusion surgery technique for the treatment of patients with LSS with DLS is still controversial.

The conventional open decompression alone surgery destroys paravertebral soft tissues, which may cause iatrogenic lumbar instability, so the fusion may need to be added. Minimally invasive (MIS) decompression surgery, especially endoscopic spinal decompression surgery, has gained increased focus in the spinal community with the notion of preserving muscular structures as basic elements for the spine and daily motions. From this perspective, minimally invasive (MIS) decompression surgery, especially endoscopic spinal decompression surgery, is becoming increasingly popular, especially in Asia [8].

Percutaneous endoscopic lumbar decompression (PELD), mainly including percutaneous transforaminal endoscopic decompression (PTED) and percutaneous interlaminar endoscopic decompression (PIED), is favored by patients with degenerative disc disease (DDD) worldwide [9, 10]. Compared with open surgery, PTED has unique advantages: less trauma, a faster recovery, lower costs, a lower rate of anesthesia-related morbidities, a lower incidence of internal fixation-related complications, and a higher percentage of satisfied patients [11, 12]. PTED has little impact on the progression of slippage, as spontaneous fusion can occur in geriatric patients during the natural course of DLS, and PTED does not damage biomechanical structures, especially the posterior ligament complex (PLC) [13, 14].

Surgeons [15] have demonstrated that the PIED technique has favorable clinical efficacy for patients with lumbar spinal stenosis and DLS; the technique resolves unilateral neurogenic claudication or radiculopathy. Salimi [16] have demonstrated that the effectiveness and radiological changes after minimally invasive lumbar decompression surgery alone observed in patients with DLS were not inferior to those of patients with LSS without a deformity. However, few studies have compared the effects of PTED and MIS-TLIF on LRS-DLS in geriatric patients. This study retrospectively analyzed the data of geriatric patients treated with minimally invasive PTED and traditional MIS-TLIF for LRS-DLS in our hospital to (1) compare the clinical outcomes of PTED and MIS-TLIF surgery in these patients; (2) determine the effects of PTED surgery on the stability of LRS-DLS; and (3) report the surgical techniques and perioperative complications of PTED surgery for LRS-DLS.

## Methods

### Demographic characteristics

We performed a retrospective review of geriatric patients who underwent PTED and MIS-TLIF from January 2017 to August 2019 by a single surgeon after the diagnosis of L4–L5 LRS-DLS. The study was approved by the institutional review board. Because this study reviewed preexisting data, the requirement for informed consent was waived. The inclusion criteria were as follows: (1) unilateral radicular leg pain or intermittent neurological claudication due to low-grade (I–II) L4–L5 LRS-DLS on X-ray, CT, and MRI; (2) the absence of improvement after conservative treatment for at least 3 months; and (3) 60 years of age or older. The exclusion criteria were as follows: (1) clinical instability symptoms (mainly back pain symptoms) or radiological instability on X-ray (> 3 mm dynamic sagittal translation); (2) prior lumbar surgery; and (3) tumor, infection, or trauma. The demographic characteristics and perioperative outcomes were reviewed. An independent surgeon evaluated the modified MacNab criteria and VAS and ODI scores. X-ray examinations were performed 1 year after surgery to assess the progression of spondylolisthesis in the PTED group and bone fusion in the MIS-TLIF group [17].

### Surgical procedures

**Fig. 1:**
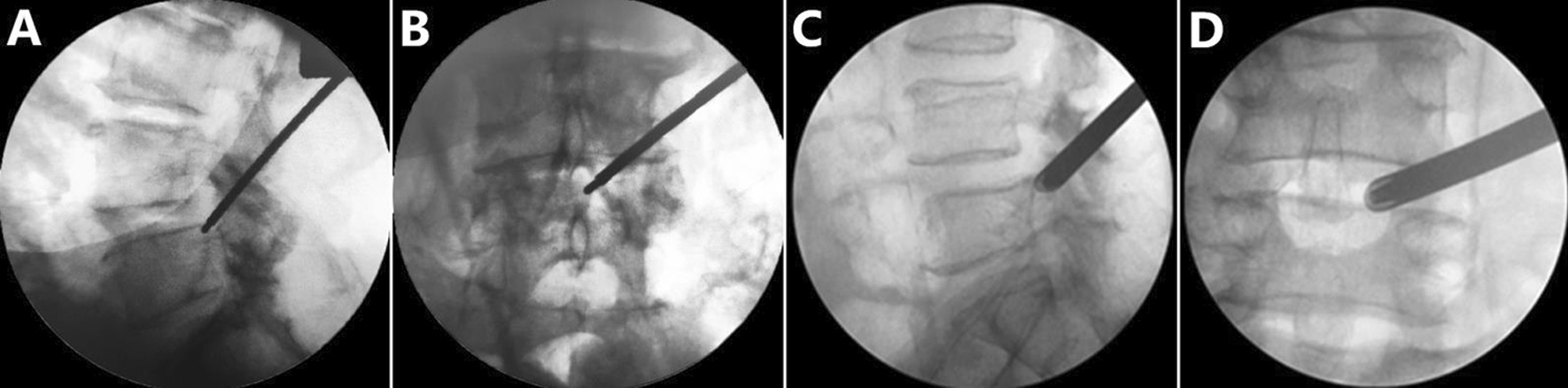
Fluoroscopic views. **A**, **B** The drill was inserted to resect the LF and the ventral osteophytes on the SAP. **C**, **D** The working cannula was placed

**Fig. 2:**
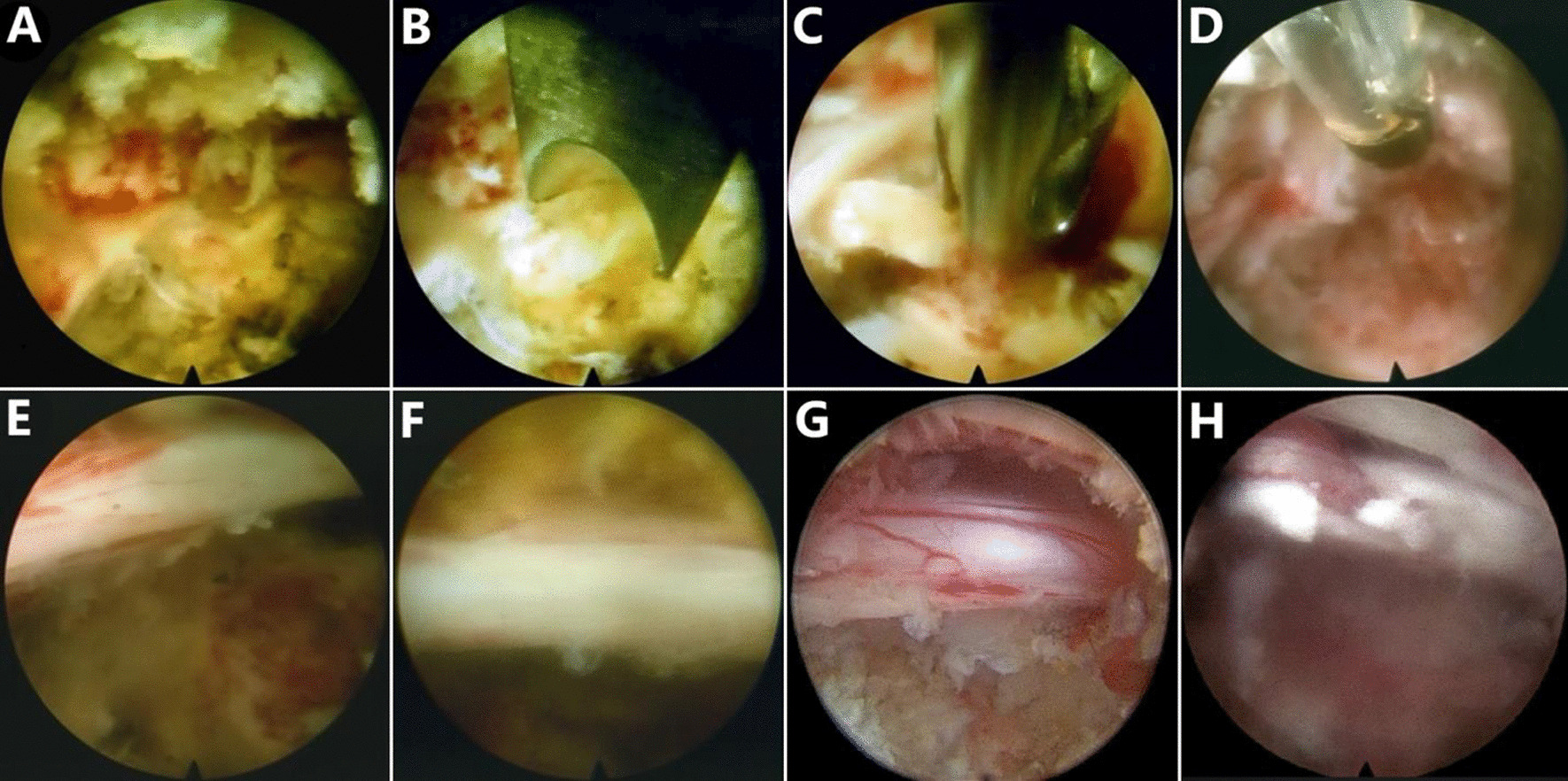
Endoscopic views. **A** Endoscopic view of the hypertrophic posterior longitudinal ligament, extruded disc material, and perineural scar. **B**–**G** After the endoscopic instruments were used to carefully remove the vertebral body, ventral decompression of the traversing nerve root (L5) was completed. **H** The dura mater was torn

**Fig. 3:**
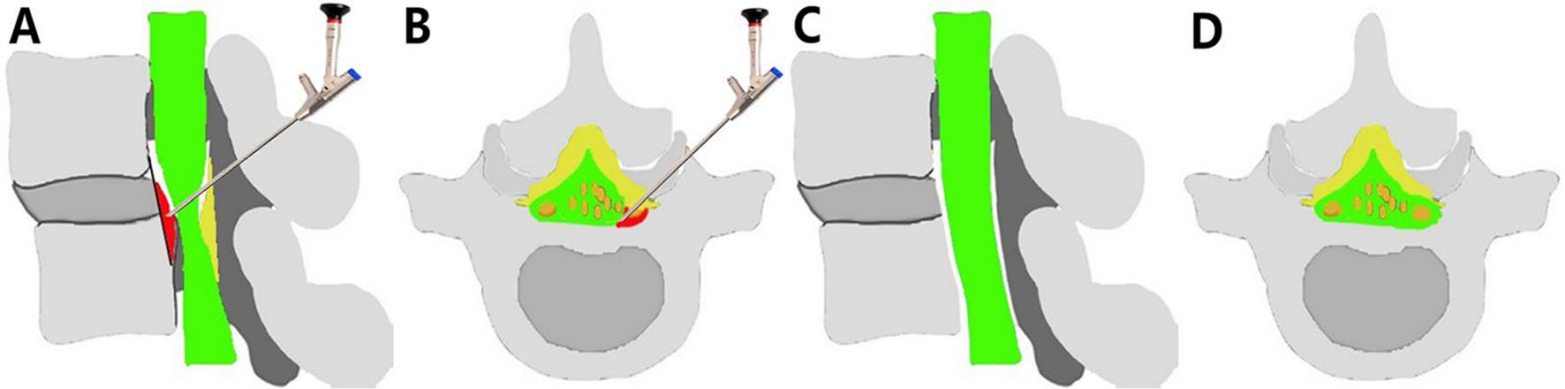
Illustrations of the 270-degree PTED. **A**, **B** Specific pathologic features of LRS-DLS. **C**, **D** Final view of the nerve 270-degree decompression status and the restoration of the lateral recess

For the PTED group, the surgical procedure (based on the L4–L5 segment of DLS) was performed following methods reported in the literature [18]. The following steps were performed: (1) part of the superior articular process (SAP) of L5 was removed. A soft pillow was placed under the patients' waist, while the patient was in the lateral decubitus position with their knee and hip flexed. The incision was located 8–12 cm from the midline horizontally and 1–3 cm above the iliac on the side with leg pain. The mixed local anesthetic, which consisted of 30 mL 1:200,000 epinephrine and 20 mL 2% lidocaine, was only used in PTED group. After 5 mL of the mixed anesthetic was inserted into the skin at the entry point, 20 mL was inserted into the trajectory, 15 mL was inserted into the articular process, and 10 mL was inserted into the foramen. Then, 0.8–1.0 cm of skin and the subcutaneous fascia were incised. Drills were used to resect the ventral osteophytes on the SAP. The PTED system (Hoogland Spine Products, Germany) was inserted (Fig. 1). (2) Parts of the ipsilateral ligamentum flavum, perineural scar, and extruded lumbar disc material were completely resected with endoscopic forceps (Fig. 2). (3) The superior endplate of the L5 vertebral body was removed by endoscopic micro punches and a bone knife. Therefore, 270-degree decompression of the traversing nerve root was achieved (Fig. 3). The drainage tube was placed after hemostasis was reached.

For the MIS-TLIF group, the surgical procedure was performed in accordance with methods reported in the literature [19]. After successful general anesthesia with tracheal intubation, the patient was placed in a prone position with chest and hip pads, and the L4–L5 intervertebral space was marked with X-ray fluoroscopy. The skin and subcutaneous fascia were cut; a trans-muscular surgical corridor was created with two micro-laminectomy retractors docking on the facet joint complex. After exposing the bony structure, part of the lamina and inferior articular process of L4 and the upper L5 articular process were removed with the rongeur on the ipsilateral side, and the hypertrophic ligamentum flavum was peeled backward. If MRI showed contralateral lateral recess stenosis, then predecompression was performed on the contralateral side. After decompression on the dorsal side, the nucleus pulposus and endplate cartilage were removed with forceps. An appropriate cage (Medtronic) filled with autograft from laminectomy was placed in the center of the intervertebral space via the Kambin’s triangle area. After adequate hemostasis was achieved, two drainage tubes were placed and removed when the drainage volume was < 50 mL/d.

Postoperatively, patients was treated with oral nonsteroidal anti-inflammatory drugs and antibiotics for 3 days. All patients were encouraged to perform straight leg raising 1 day postoperatively, and moderate off-bed activity with a brace 2–3 days postoperatively. On the third postoperative day, patients were allowed to go home if their lower extremity pain symptoms were effectively relieved with no evidence of infection. The patient demographics and perioperative outcomes were compared. The VAS score, ODI, and modified Macnab criteria were used to evaluate the clinical outcomes [20].

### 3Statistical analysis

The SPSS 25 program (IBM Corporation, USA) was used to perform statistical analysis. Repeated-measures analysis of variance was used to compare the VAS and ODI scores between the two groups. The independent-samples *t* test and Mann–Whitney *U* test or Fisher’s exact test were used to assess the demographic characteristics and the perioperative outcomes. The level of statistical significance was set at *P* < 0.05.

## Results

### Demographic characteristics and perioperative outcomes

Of the 90 patients who met the study inclusion criteria, 44 underwent PTED and 46 underwent MIS-TLIF. The baseline parameters are given in Table 1. The surgical parameters, including the operative time, blood loss, incision length, drainage time, drainage volume, length of hospital stay and number of complications, are shown in Table 2. The perioperative outcomes in the patients who underwent PTED were remarkably better than in those who underwent MIS-TLIF.

**Table 1:** Preoperative demographic characteristics

Characteristics	PTED Group (*n* = 44)	MIS-TLIF Group (*n* = 46)	*P* Value
Age (years)	70.3 ± 7.5	68.6 ± 6.0	0.250
Sex (male/female)	11/33	13/33	0.727
Duration of symptoms (months)	29.75 ± 31.32	27.09 ± 29.17	0.677
Comorbidities (yes/no)	11/33	12/34	0.906
Side (right/left)	21/23	24/22	0.673
Slippage grade (I/II)	29/15	29/17	0.776

**Table 2:** Perioperative outcomes

Characteristics	PTED (*n* = 44)	MIS-TLIF (*n* = 46)	*P* Value
Duration of surgery (minutes)	71.36 ± 14.72	128.02 ± 19.02	0.00
Estimated blood loss (ml)	12.2 ± 5.37	361.96 ± 168.39	0.00
Incision length (cm)	1.14 ± 0.24	8.39 ± 0.98	0.00
drainage time (d)	1.05 ± 0.21	4.89 ± 1.12	0.00
Drainage volume (ml)	26.27 ± 30.40	563.59 ± 148.21	0.00
Hospital stay (day)	5.54 ± 1.58	12.02 ± 4.92	0.00
Complications (yes/no)	3/41	6/40	0.527
Interbody fusion (yes/no)	–	45/1	–
Slippage grade (I/II)	28/16	–	–

### Clinical results

**Fig. 4:**
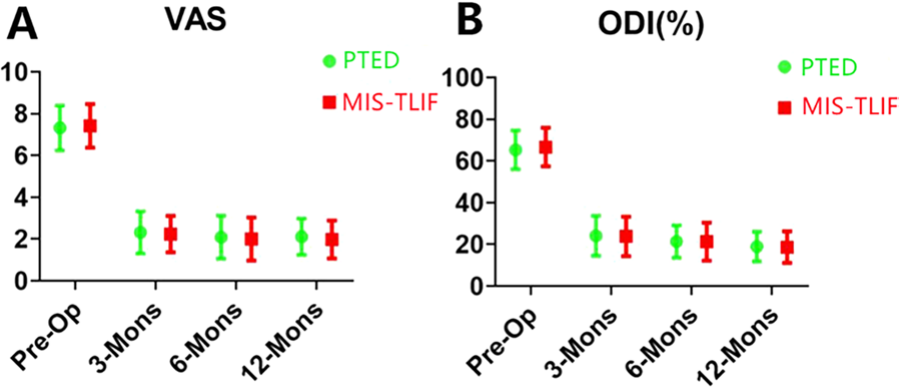
Clinical outcomes at different follow-up time points. **A** VAS score for leg pain in both groups. **B** ODI score for both groups

Preoperatively, the mean VAS and ODI scores were similar between the two groups (7.32 ± 1.07 vs. 7.41 ± 1.05, *P* > 0.05; 65.32 ± 9.29 vs. 66.65 ± 9.23, *P* > 0.05). At 12 months, we observed similar improvements in the mean VAS score for leg pain and ODI score in the PTED and TLIF groups (Fig. 4). Moreover, there were no differences between the groups at any follow-up time point (*P* > 0.05). Based on the modified MacNab criteria, the good-to-excellent rate was 90.9% (40/44) in the PTED group and 91.3% (42/46) in the MIS-TLIF group at the final follow-up. During the 1-year follow-up in the PTED group, the Meyerding grade of spondylolisthesis changed from grade I to grade II in one case, but the clinical effect was satisfactory.

### Complications

A total of three patients in the PTED group had perioperative complications. One patient had weakened ankle extensor muscle strength during drilling and was given nutrients for nerves and drugs for dehydration. The level of muscle strength had returned to normal 1 month after surgery. In one patient, when the superior endplate of the L5 vertebral body was removed with the endoscopic bone knife and micro punches, the extent of visual field bleeding was severe. The dura mater was torn when the radiofrequency electrocoagulation was used to stop the bleeding (Fig. 2). The patient complained of neck pain, and the heart rate increased, so the operation was suspended. The process was continued after the neck pain disappeared. The neck pain recurred within a short period, and the patient returned to the ward after his condition stabilized. The patient was instructed to lie supine, and 120 mL of pink liquid was drained within 24 h after surgery. When 50 mL of clear liquid was drained within 72 h postoperatively, the drainage tube was removed. One patient had unsatisfactory relief, and nerve function returned to normal after the reoperation. No incision infections, cardiovascular or cerebrovascular accidents or other complications occurred.

Complications occurred in 6 patients in the MIS-TLIF group. One patient developed an epidural hematoma after the operation. The function of the lower limbs, stool and urination decreased progressively after the procedure. The epidura hematoma was removed by emergency surgery after MRI. Two patients had postoperative wound infections, one of whom had postoperative hypostatic pneumonia. The incision healed after debridement and sensitive antibiotics. In one patient, the dura mater was torn during lamina removal, and the dural sac was sutured under a microscope. There was no cerebrospinal fluid leakage after the operation. One patient had a cerebrovascular incident and cerebrovascular interventional treatment; the patient’s condition improved without severe sequelae. Only one patient did not exhibit intervertebral fusion by the 1-year follow-up, but the patient's lower extremity symptoms were relieved satisfactorily, no reoperation was performed. There were no reports of other postoperative complications during the follow-up.

## Discussion

Based on the anatomic and clinical manifestations, DLS can be divided into three categories [21]. One category includes cases whose back pain is accompanied with lumbar instability. The leading cause of this pathologic disorder is the degeneration of intervertebral discs and facet joints. Back pain does not progress beyond the back and the knee joint. Another category includes cases in which intermittent claudication with central spinal stenosis. Intermittent claudication is caused by cauda equina nerve ischemia after overactivity. The last category, lateral recess stenosis of the DLS (LRS-DLS), which is the subject of this research, mainly manifests as radiculopathy [22]. Such cases are mainly caused by stenosis of the lateral recess and intervertebral foramina, which compress the L5 nerve root (Fig. 3). The LF on the dorsal side of the nerve root, the lateral hypertrophic SAP, the ventral intervertebral disc, the protruding posterior disc and posterior longitudinal ligament, and particularly, the superior endplate of the L5 vertebral body, which are magnified in the presence of degenerative slip, work together to compress the traversing nerve root (L5) [23]. In this retrospective research, we tested the hypothesis that the effectiveness of 270-degree PTED under local anesthesia is noninferior to MIS-TLIF in geriatric patients with LRS-DLS regarding the VAS score, ODI score and modified MacNab criteria. Furthermore, PTED was expected to be advantageous in terms of morbidities and the extent of trauma.

Most experts believe that surgical intervention can be used if nonsurgical treatment fails after 3–6 months [24]. The purposes of conventional open lumbar spinal surgery include decompression of the nerve roots and deformity correction. Traditional open surgery includes laminectomy with or without interbody fusion. Karsy [25] believed that compared with conservative treatment, laminectomy leads to better lower limb function and better cost-effectiveness ratio. Some researchers [26] believe that decompression surgery alone can not only reduce the cost of treatment but also shorten the operation and hospitalization times, while the effect is equivalent to fusion surgery. However, other researchers have confirmed that laminectomy and the destruction of articular processes by more than 50% can cause iatrogenic destabilization, which leads to reoperation [27]. Although fusion surgery overcomes the disadvantages of laminectomy and has demonstrated favorable results, fusion is time-consuming and is associated with a risk of nerve root damage [28].

For geriatric DLS patients, especially with comorbidities, the probability of perioperative accidents may be high and a simplified spinal surgery protocol is expected. For elderly patients with osteoporosis, complications such as compression fractures of the adjacent vertebral body, loosening of internal nails, adjacent segment disease may occur. The additional presence of diabetes or poor nutritional status can lead to delayed incision healing. For patients with severe respiratory diseases and patients who cannot tolerate general anesthesia, traditional open surgery may not be suitable. Therefore, surgeons have considered whether there is an ideal microinvasive surgery to resolve the symptoms of DLS in elderly patients without destroying the stability of the spine [29, 30].

**Fig. 5:**
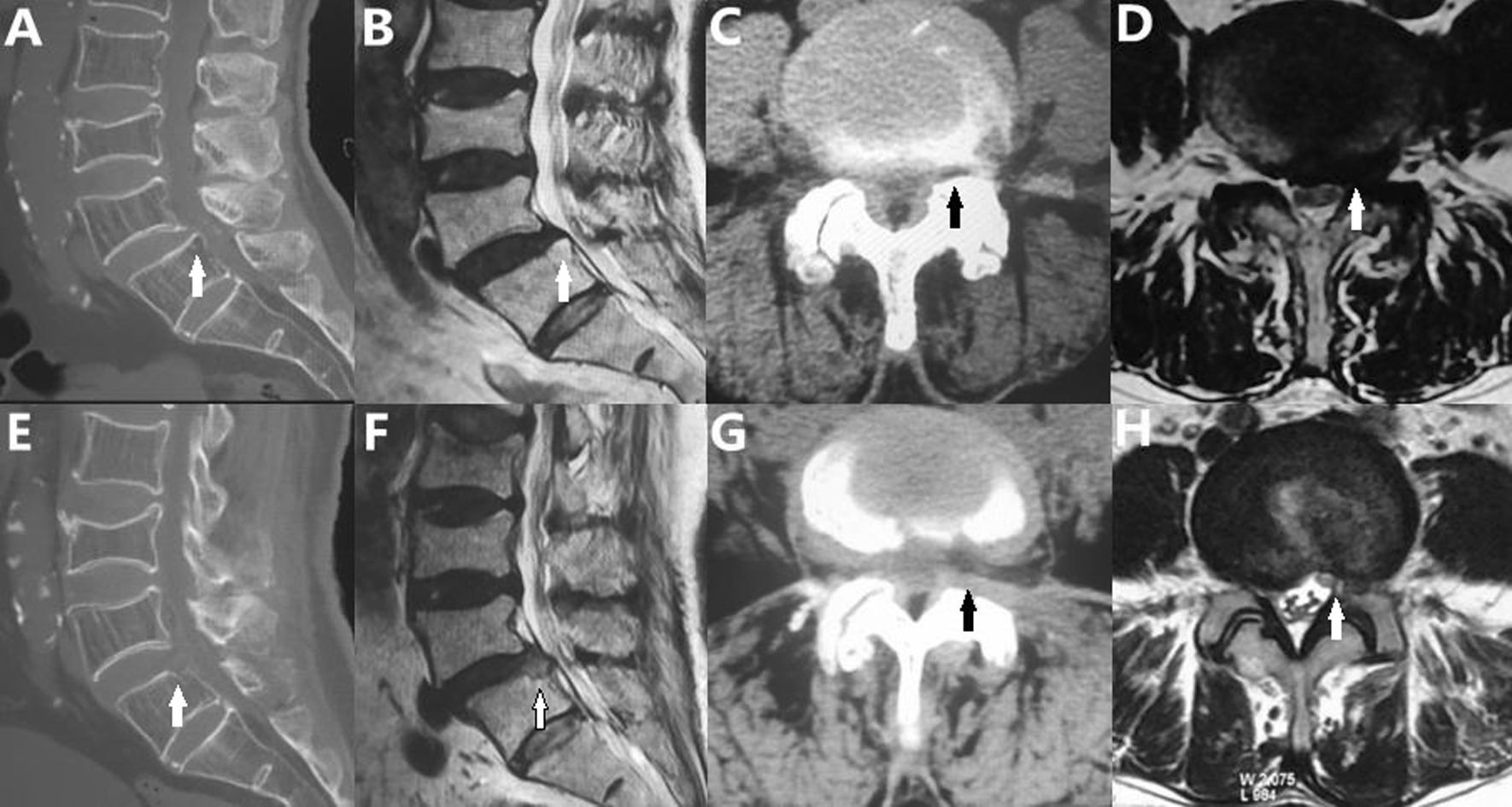
Pre- and postoperative CT and MRI. **A**–**D** The superior endplate of L5 before surgery and lateral recess stenosis. **E**, **F** The protruded vertebral bone was removed, and the original spinal canal shape was restored. The ventral and dorsal edges of the spinal canal became smooth and continuous. **G**, **H** The lateral recess was enlarged

PTED is less invasive than minimally invasive decompression for geriatric patients with degenerative spinal disease [31]. Some studies used PTED to treat DLS with unilateral radicular symptoms and demonstrated positive clinical results, but the comparison with MIS-TLIF has not been reported [32]. For complete decompression of the nerve root, surgeons use a bone drill to abrade part of the articular process to expand the intervertebral foramen and use a microscopic bone knife, the microscopic drill and nucleus forceps to remove the bone of the superior endplate of the L5 vertebral body (Fig. 5). Both ends of the lateral recess stenosis should be thoroughly explored to expand the area of lateral recess stenosis. The operation does not cause severe damage to the ligament complex behind the L4–L5 vertebral bodies, and the facet joint is not resected excessively. Studies have shown that the natural course of DLS tends to be stable [33]. Besides, for geriatric patients, the main etiology of lateral recess stenosis associated with L4–L5 degenerative lumbar spondylolisthesis is nerve root compression by tissues such as the superior endplate of the L5 vertebral body, which can be decompressed using percutaneous transforaminal endoscopic decompression and can relieve pain in patients. The destruction of the articular processes is minimal in PTED, so the operation does not lead to obvious iatrogenic destabilization theoretically, thus avoiding fusion surgery.

During the 1-year follow-up in the PTED group, the Meyerding grade of spondylolisthesis changed from grade I to grade II in one case, but the clinical effect was satisfactory. For surgeons and most elderly patients with DLS, the only use of local anesthesia is ideal [34]. If the surgical equipment stimulates the nerve root during the operation, the awake patient will experience abnormal sensation, and the surgeon can stop the process timely. The local anesthesia reduces the occurrence of complications related to general anesthesia in elderly patients. The patient can be asked whether he or she subjectively feels the symptoms have been alleviated, and the straight-leg elevation test can be performed; these responses can be used to determine whether the operation should be terminated (e.g., a negative straight-leg elevation test result). Due to the long-term oral administration of anticoagulants, the extent of intraoperative visual field bleeding was severe in some patients. After lidocaine combined with epinephrine hydrochloride solution was administered, the visual field was clearer without obvious drug-related complications. The water pressure of the lavage fluid can also be appropriately reduced, theoretically reducing the incidence of spinal hypertension reactions [35].

Six patients in the MIS-TLIF group had complications, which may be related to the operation itself.

Three patients in the PTED group had complications. The findings were as follows: one patient's ankle dorsi extensor strength decreased when drilling in the foramen was performed; after functional exercises, the level of muscle strength gradually returned to normal. For geriatric patients with DLS, severe intervertebral disc degeneration, reduced intervertebral space height, and severe articular process hyperplasia lead to a reduction in the Kambin’s triangle area. When the bone drill is grinding the upper articular of L5, the bone drill angle must be adjusted, which can lead to L4 nerve root injury. To avoid L4 nerve root injury, the head angle of the bone drill should be restricted, and the patient's response should be observed to ensure that the working channel is accurately established. If the strength of the ankle dorsiflexor muscles decreases, the drill should be withdrawn timely.

One patient had a spinal hypertension reaction during the operation [36]. Spinal hypertension reactions have various manifestations, including neck pain, tinnitus, visual impairment, epilepsy, increased blood pressure, an increased heart rate, limb sensation and movement disorders, and perineal foreign body sensation. We speculate that, due to the patient's long-term use of anticoagulant drugs, the extent of bleeding was severe during the surgical removal of the upper edge of the L5 vertebral body. The visual field was not clear, and the dural sac was cut by the device. To ensure a clear view, the irrigation pressure was increased. Then, the fluid entered the spinal canal along with the torn dura mater and mixed with the cerebrospinal fluid, resulting in an acute increase in pressure within the spinal cord. To avoid this phenomenon, the following points should be considered: during the operation, the head of the patient should be elevated; the water irrigation pressure should not be too high; to ensure safety, the operation time should be as short as possible; and the use of local anesthetics and adrenal hydrochloride may reduce bleeding in the visual field and reduce the pressure of the lavage fluid.

One patient did not exhibit significant relief of radiating pain in the left lower extremity after the operation. After reviewing the intraoperative fluoroscopy results, we found that the cannula did not reach the midline of the spine channel, and we failed to completely remove the bone of the posterior upper edge of the L5 vertebral body which compressed the nerve root. After the second operation, the patient's symptoms were relieved satisfactorily. Therefore, during the operation, the channel should be established as accurately as possible to avoid reducing the space for the operation under the microscope and increasing the difficulty of complete decompression.

The study has some limitations: (1) Because the inclusion criteria for this research were strict, the number of patients included was relatively small. (2) Long-term follow-up data are needed, as both PTED and MIS-TLIF show good short-term results. (3) The cross-sectional area of the lateral recess was not measured, as we attributed the relief of uniliteral leg symptoms to the enlargement of the lateral recess.

## Conclusion

Both PTED and MIS-TLIF showed favorable outcomes for the treatment of single-level LRS-DLS in geriatric patients. In terms of the clinical results and perioperative quality of life, 270-degree PTED under local anesthesia can be considered an effective supplement to MIS-TLIF for select geriatric patients. However, prospective randomized controlled trials (RCTs) with larger sample sizes and longer follow-up periods are required in the future.

## Data Availability

The data used to support the findings of this study are available from the corresponding author upon request.

## Abbreviations

PTED: Percutaneous transforaminal endoscopic decompression
DLS: Degenerative lumbar spondylolisthesis
DDD: Degenerative disc disease
LRS: Lateral recess stenosis
MIS-TLIF: Minimally invasive transforaminal lumbar interbody fusion
VAS: The visual analog scale
ODI: Oswestry Disability Index
MED: Microendoscopic decompression
SAP: Superior articular process
PLC: Posterior ligament complex

## References

[1] Junghanns H (1930). Spondylolisthesen ohne Spalt in Zwischengelenkstueck. Arch Orthop Unfallchir.

[2] Badhiwala JH, Leung SN, Jiang F (2020). In-hospital course and complications of laminectomy alone versus laminectomy plus instrumented posterolateral fusion for lumbar degenerative spondylolisthesis: a retrospective analysis of 1804 patients from the NSQIP Database. Spine.

[3] Gaab MR (2018). Transforaminal endoscopic decompression in lumbar spondylolisthesis background and perspectives. World Neurosurg.

[4] Gadjradj PS, Basilious M, Goldberg JL, et al. Decompression alone versus decompression with fusion in patients with lumbar spinal stenosis with degenerative spondylolisthesis: a systematic review and meta-analysis. Eur Spine J. 2023 Jan 6.Epub ahead of print. 10.1007/s00586-022-07507-1 36609887

[5] Ghogawala Z, Dziura J, Butler WE (2016). Laminectomy plus fusion versus laminectomy alone for lumbar spondylolisthesis. N Engl J Med.

[6] Försth P, Ólafsson G, Carlsson T (2016). A randomized, controlled trial of fusion surgery for lumbar spinal stenosis. N Engl J Med.

[7] Austevoll IM, Hermansen E, Fagerland MW (2021). Decompression with or without fusion in degenerative lumbar spondylolisthesis. N Engl J Med.

[8] Zhang J, Liu TF, Shan H (2021). Decompression using minimally invasive surgery for lumbar spinal stenosis associated with degenerative spondylolisthesis: a review. Pain Ther.

[9] Yu P, Zan P, Zhang X (2021). Comparison of percutaneous transforaminal endoscopic discectomy and micro endoscopic discectomy for the surgical management of symptomatic lumbar disc herniation: a multicenter retrospective cohort study with a minimum of 2 years' follow-up. Pain Physician.

[10] Telfeian AE, Syed S, Oyelese A (2020). Endoscopic surgical resection of the retropulsed S1 vertebral endplate in L5–S1 spondylolisthesis: case series. Pain Physician.

[11] Gadjradj PS, Harhangi BS, Amelink J (2020). Percutaneous transforaminal endoscopic discectomy versus open microdiscectomy for lumbar disc herniation: a systematic review and meta-analysis. Spine.

[12] Gadjradj PS, Rubinstein SM, Peul WC (2022). Full endoscopic versus open discectomy for sciatica: randomised controlled non-inferiority trial. BMJ.

[13] Wu Q, Yuan S, Fan N (2021). Clinical outcomes of percutaneous endoscopic lumbar discectomy for the treatment of grade I and grade II degenerative lumbar spondylolisthesis: a retrospective study with a minimum five-year follow-up. Pain Physician.

[14] Hasegawa K, Kitahara K, Shimoda H (2014). Lumbar degenerative spondylolisthesis is not always unstable: clinicobiomechanical evidence. Spine.

[15] Sriphirom P, Siramanakul C, Chaipanha P (2021). Clinical outcomes of interlaminar percutaneous endoscopic decompression for degenerative lumbar spondylolisthesis with spinal stenosis. Brain Sci.

[16] Salimi H, Toyoda H, Terai H (2022). Mid-term changes in spinopelvic sagittal alignment in lumbar spinal stenosis with coexisting degenerative spondylolisthesis or scoliosis after minimally invasive lumbar decompression surgery: minimum five-year follow-up. Spine J.

[17] Wei FL, Zhou CP, Gao QY (2022). Decompression alone or decompression and fusion in degenerative lumbar spondylolisthesis. EClinicalMedicine.

[18] Cheng XK, Chen B (2020). Percutaneous transforaminal endoscopic decompression for geriatric patients with central spinal stenosis and degenerative lumbar spondylolisthesis: a novel surgical technique and clinical outcomes. Clin Interv Aging.

[19] Chan AK, Bydon M, Bisson EF (2023). Minimally invasive versus open transforaminal lumbar interbody fusion for grade I lumbar spondylolisthesis: 5-year follow-up from the prospective multicenter quality outcomes database registry. Neurosurg Focus.

[20] Macnab I (1971). Negative disc exploration: an analysis of the causes of nerve-root involvement in sixty-eight patients. J Bone Joint Surg Am.

[21] Kirkaldy-Willis WH, Wedge JH, Yong-Hing K (1978). Pathology and pathogenesis of lumbar spondylosis and stenosis. Spine.

[22] Dijkerman ML, Overdevest GM, Moojen WA (2018). Decompression with or without concomitant fusion in lumbar stenosis due to degenerative spondylolisthesis: a systematic review. Eur Spine J.

[23] Rampersaud YR, Fisher C, Yee A (2014). Health-related quality of life following decompression compared to decompression and fusion for degenerative lumbar spondylolisthesis: a Canadian multicentre study. Can J Surg.

[24] Matz PG, Meagher RJ, Lamer T (2016). Guideline summary review: an evidence-based clinical guideline for the diagnosis and treatment of degenerative lumbar spondylolisthesis. Spine J.

[25] Karsy M, Bisson EF (2019). Surgical versus nonsurgical treatment of lumbar spondylolisthesis. Neurosurg Clin N Am.

[26] Vorhies JS, Hernandez-Boussard T, Alamin T (2018). Treatment of degenerative lumbar spondylolisthesis with fusion or decompression alone results in similar rates of reoperation at 5 years. Clin Spine Surg.

[27] Urakawa H, Jones T, Samuel A (2020). The necessity and risk factors of subsequent fusion after decompression alone for lumbar spinal stenosis with lumbar spondylolisthesis: 5 years follow-up in two different large populations. Spine J.

[28] Oikonomidis S, Meyer C, Scheyerer MJ (2020). Lumbar spinal fusion of low-grade degenerative spondylolisthesis (Meyerding grade I and II): do reduction and correction of the radiological sagittal parameters correlate with better clinical outcome?. Arch Orthop Trauma Surg.

[29] Chan AK, Bisson EF, Bydon M (2020). A comparison of minimally invasive and open transforaminal lumbar interbody fusion for grade 1 degenerative lumbar spondylolisthesis: an analysis of the prospective quality outcomes database. Neurosurgery.

[30] Aihara T, Endo K, Suzuki H (2021). Long-term outcomes following lumbar microendoscopic decompression for lumbar spinal stenosis with and without degenerative spondylolisthesis: minimum 10-year follow-up. World Neurosurg.

[31] Hasan S, McGrath LB, Sen RD (2019). Comparison of full-endoscopic and minimally invasive decompression for lumbar spinal stenosis in the setting of degenerative scoliosis and spondylolisthesis. Neurosurg Focus.

[32] Cheng XK, Cheng YP, Liu ZY (2020). Percutaneous transforaminal endoscopic decompression for lumbar spinal stenosis with degenerative spondylolisthesis in the elderly. Clin Neurol Neurosurg.

[33] Matsunaga S, Sakou T, Morizono Y (1990). Natural history of degenerative spondylolisthesis: pathogenesis and natural course of the slippage. Spine.

[34] Youn MS, Shin JK, Goh TS (2018). Endoscopic posterior decompression under local anesthesia for degenerative lumbar spinal stenosis. J Neurosurg Spine.

[35] Cho JY, Lee SH, Lee HY (2011). Prevention of development of postoperative dysesthesia in transforaminal percutaneous endoscopic lumbar discectomy for intracanalicular lumbar disc herniation: floating retraction technique. Minim Invasive Neurosurg.

[36] Sairyo K, Matsuura T, Higashino K (2014). Surgery related complications in percutaneous endoscopic lumbar discectomy under local anesthesia. J Med Invest.

